# Supplementary material for: In Vivo Transmigrated Human Neutrophils Are Highly Primed for Intracellular Radical Production Induced by Monosodium Urate Crystals
Source: Int J Mol Sci. 2020 May 26;21(11):3750. doi: 10.3390/ijms21113750 (PMC7312864; doi:10.3390/ijms21113750)
Supplement: Supplementary file 1 [file ijms-21-03750-s001.zip › Suppl Figure 1.pptx]

## Slide 1
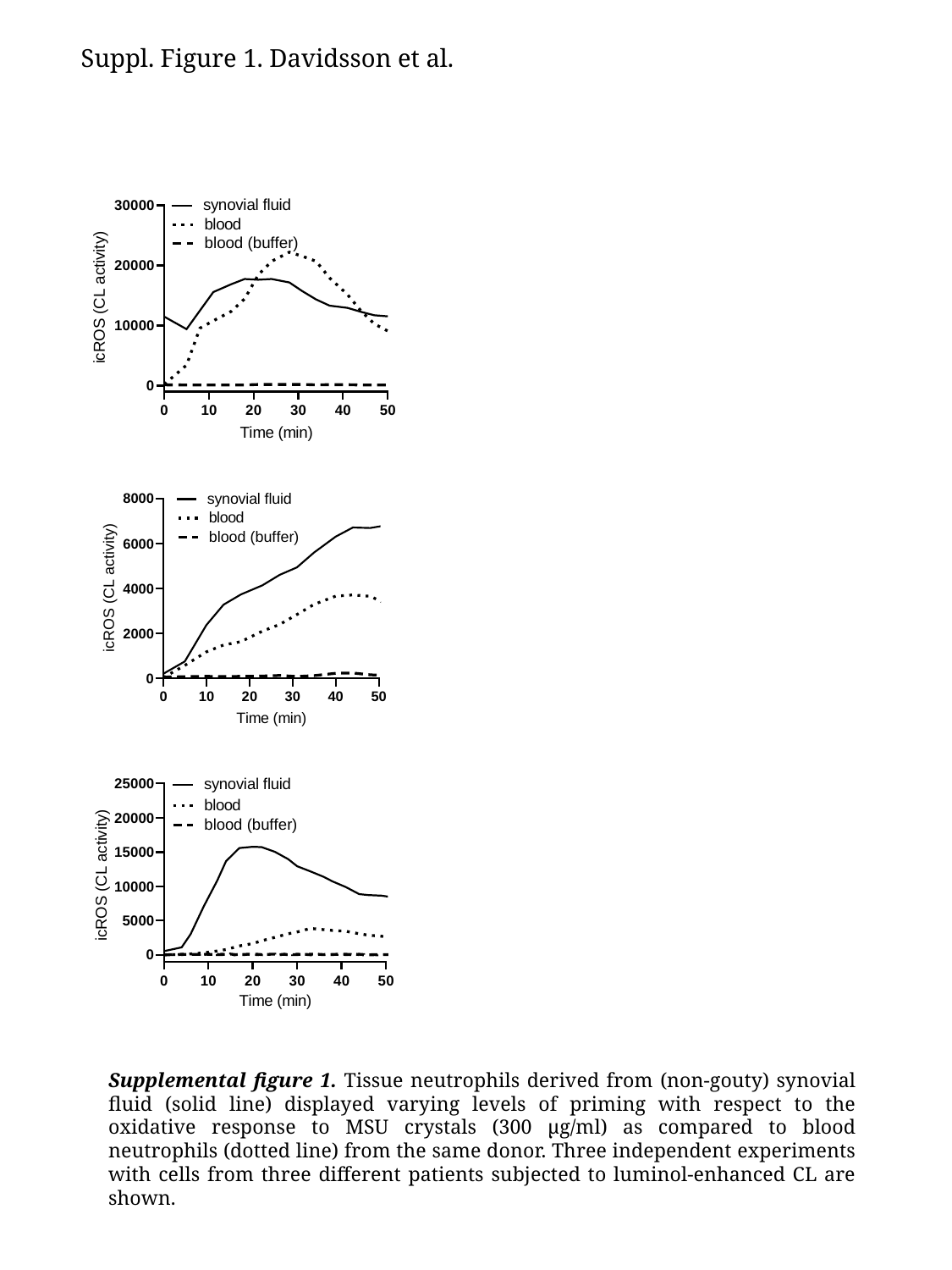

Suppl. Figure 1. Davidsson et al.
Supplemental figure 1. Tissue neutrophils derived from (non-gouty) synovial fluid (solid line) displayed varying levels of priming with respect to the oxidative response to MSU crystals (300 µg/ml) as compared to blood neutrophils (dotted line) from the same donor. Three independent experiments with cells from three different patients subjected to luminol-enhanced CL are shown.
